# Retraction Note: *Tridax procumbens* flavonoids promote osteoblast differentiation and bone formation

**DOI:** 10.1186/s40659-024-00525-x

**Published:** 2024-06-24

**Authors:** Md. Abdullah Al Mamun, Mohammad Jakir Hosen, Kamrul Islam, Amina Khatun, M. Masihul Alam, Md. Abdul Alim Al-Bari

**Affiliations:** 1 Department of Genetic Engineering and Biotechnology, Shahjalal University of Science and Technology, Sylhet, 3114 Bangladesh; 2 Department of Anthropology, Shahjalal University of Science and Technology, Sylhet, 3114 Bangladesh; 3 Department of Applied Nutrition and Food Technology, Islami University, Kustia, 7003 Bangladesh; 4 Department of Pharmacy, Rajshahi University, Rajshahi, 6205 Bangladesh

**Retraction Note: Biol Res (2015) 48:65**

10.1186/s40659-015-0056-1

The Editor-in-Chief has retracted this article because, while the article states that the experimental procedures were reviewed and approved by an ethics committee, the authors have since confirmed that ethics approval was not in place at the time that this work was conducted.

Md. Abdullah Al Mamun did not explicitly state whether they agree or disagree with this retraction. The remaining authors did not respond to correspondence from the publisher about this retraction.

